# Case Report and Literature Review: Diagnosis, Tailored Genetic Counseling and Cancer Prevention for a Locally Advanced dMMR/MSI-H/TMB-H Lung Cancer Patient With Concurrent Lynch Syndrome Mediated by a Rare *PMS2* Splicing Variant (c.1144+1G>A)

**DOI:** 10.3389/fgene.2021.799807

**Published:** 2022-01-18

**Authors:** Quanli Han, Si Liu, Zhi Cui, Qi Wang, Tonghui Ma, Liwen Jiang, Xiaomo Li, Guanghai Dai

**Affiliations:** ^1^ Department of Medical Oncology, the First Medical Center of Chinese People’s Liberation Army (PLA) General Hospital, Beijing, China; ^2^ Genetron Health (Beijing) Technology, Co. Ltd., Beijing, China

**Keywords:** Lynch syndrome, lung cancer, PMS2, splicing variant, incomplete penetrance

## Abstract

Lynch syndrome (LS) is a cancer-predisposing genetic disease mediated by pathogenic mutations in DNA mismatch repair (MMR) genes *MLH1*, *MSH2*, *MSH6*, and *PMS2*. Accumulating evidence demonstrates that there is significant biological heterogeneity across MMR genes. Compared to *MLH1* and *MSH2*, *PMS2* variant carriers have a much lower risk for LS-related cancers. Tumors in *MLH1* and *MSH2* variant carriers often display MMR deficiency (dMMR) and/or high microsatellite instability (MSI-H), two predictive biomarkers for immunotherapy efficacy. However, tumors in *PMS2* variant carriers are largely microsatellite stable (MSS) instead of MSI. Therefore, the optimal management of cancer patients with LS requires the integration of disease stage, MMR gene penetrance, dMMR/MSI status, and tumor mutational burden (TMB). In this work, we presented a locally advanced lung cancer patient with dMMR/MSI-H/TMB-H tumor and selective loss of PMS2 by immunohistochemistry. Germline testing revealed a rare *PMS2* splicing variant (c.1144+1G>A) in the proband and his healthy daughter. The diagnosis of LS was made based on genetic analysis of this variant and literature review. Given the incomplete penetrance of *PMS2*, the proband and the carrier received tailored genetic counseling. To reduce cancer risk, the proband received four cycles of nivolumab plus chemotherapy and achieved a disease-free survival of sixteen months.

## Introduction

In the past decade, cancer immunotherapy has shifted the landscape of cancer treatment (Tang et al., 2018). Predictive biomarkers such as PD-L1 expression level have greatly facilitated the selection of patients for immunotherapy in some cancer types (Doroshow et al., 2021). In 2017, FDA approved PD-1 antibody pembrolizumab to treat patients with unresectable or metastatic mismatch repair deficiency (dMMR) and/or microsatellite instability-high (MSI-H) solid tumors, making it the first tissue/site-agnostic predictive biomarker (Marcus et al., 2019).

MMR deficiency is mediated by somatic or germline mutations in MMR genes (*MLH1*, *MSH2*, *MSH6*, and *PMS2*) and rarely, *EPCAM* (Le et al., 2015). Pathogenic germline variants in MMR genes cause Lynch Syndrome (LS), a genetic disease predisposing patients to multiple types of cancers (Lynch et al., 2015). Therefore, the diagnosis of LS is essential for the treatment and cancer-risk reduction for LS patients and family members harboring pathogenic variants (Yurgelun and Hampel, 2018).

The optimal testing and treatment of LS patients require a good knowledge of the pan-cancer prevalence and heterogeneity of pathogenic MMR gene variants. To explore the prevalence of LS across solid tumors according to MSI status, researchers at the Memorial Sloan Kettering Cancer Center (MSKCC) screened 15,045 patients of more than 50 cancer types (Latham et al., 2019). Among LS patients with MSI tumors, half had tumors other than colorectal and endometrial cancer, including gastric, pancreas, small bowel, and germ cell tumors. However, none of the 1,952 lung cancer patients had LS. While most tumors from *MLH1* and *MSH2* carriers are MSI, more than two-thirds of tumors from *PMS2* carriers are microsatellite stable (MSS) (Latham et al., 2019).

Recent studies including the Prospective Lynch Syndrome Database (PLSD) and the International Mismatch Repair Consortium revealed that MMR gene variants had distinct penetrance (Dominguez-Valentin et al., 2020; Dominguez-Valentin et al., 2021; International Mismatch Repair Consortium, 2021). Pathogenic *MLH1* and *MSH2* variants caused high penetrance in a broad spectrum of LS cancers, while pathogenic *PMS2* variants were associated with low penetrance in few LS-related cancers. Therefore, the cancer screening, chemoprevention, and risk-reduction surgery protocol for carriers of *MLH1* and *MSH2* variants should be tailored to carriers of *PMS2* variants (Balmaña et al., 2013; Stoffel et al., 2015; Gupta et al., 2019; Seppälä et al., 2021a). Here, we presented the diagnosis, tailored genetic counseling, and cancer prevention of a locally advanced lung cancer patient with dMMR/MSI-H/TMB-H tumor and *PMS2*-LS.

## Case Report and Genetic Analysis

**FIGURE 1:**
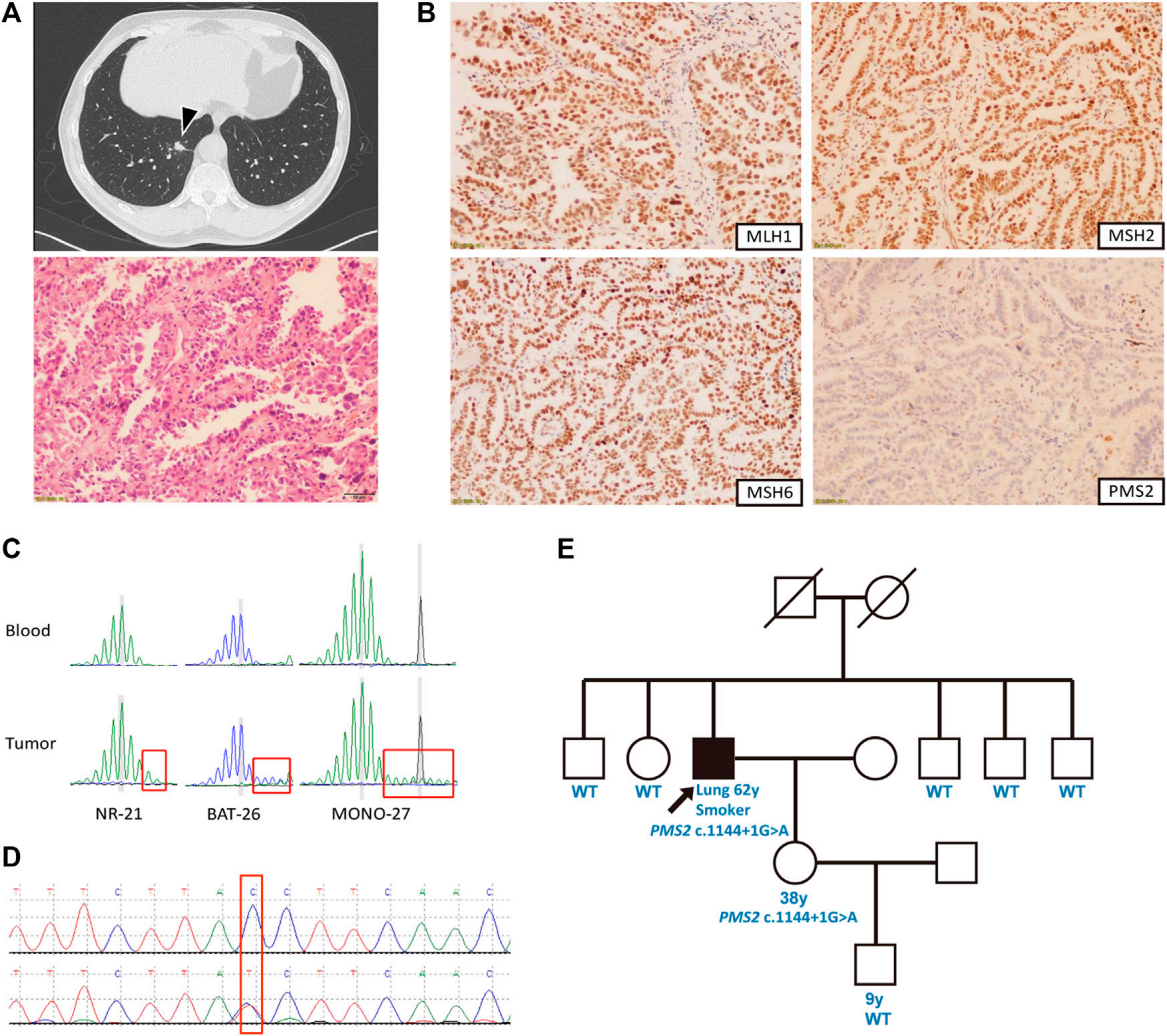
Case summary. **(A)** Clinical findings of the patient. Above: Chest computed tomography revealing a mass in the right lower lobe (arrow); below: Hematoxylin and eosin-stained tumor tissue sections presenting adenocarcinoma (magnification, ×400). **(B)** Immunohistochemical staining showing absence of PMS2 as well as presence of MLH1, MSH2 and MSH6 in the tumor cell nuclei. **(C)** Capillary electrophoresis results showed loss of stability of three microsatellite biomarkers in tumor compared to the blood control, indicating high microsatellite instability. **(D)** Sequence chromatogram of the patient’s daughter containing the same *PMS2* c.1144+1G>A mutation. **(E)** Pedigree of the patient’s family. The proband is indicated with an arrow and black denotes the cancer-affected individual.

Our proband was a 62-year-old Chinese man with 40-pack-year smoking history, presented with a consolidation shadow in the lower lobe of the right lung discovered by chest computed tomography (CT) (Figure 1A). The CT scan also detected local occlusion of internal small bronchus, subpleural nodular ground-glass opacity on the posterior segment of the right superior lobe, as well as a nodular contour in the right adrenal gland. He underwent thoracoscopic right lower lobectomy and mediastinal lymphadenectomy. Postoperative pathology showed a stage IIIA lung adenocarcinoma (T1aN2M0) with regional lymph node metastases, which had a size of 0.9 × 0.6 × 0.6 cm. Genomic profiling with a multi-gene next-generation sequencing (NGS) panel (Onco Panscan™, Genetron Health) showed a *KRAS* G13D mutation, a *TP53* R267W mutation, and a high tumor mutational burden (TMB-H, 13.62 mutations/Mb) (Table 1). PD-L1 immunochemistry (IHC), MMR IHC, and microsatellite instability (MSI) testing revealed that his tumor was PD-L1-positive (TPS 8%), MSI-H, and dMMR (Figures 1B,C).

**TABLE 1:** Somatic and germline testing results

Mutation type	Gene	Nucleotide change	Amino acid change	Mutation effect	VAF (%)
Germline	*PMS2*	c.1144+1G>A		Splicing	
Somatic	*KRAS*	c.38G>A	p.Gly13Asp	Nonsynonymous	20.9
	*ARID1A*	c.4555dup C	p.Gln1519ProfsTer13	Frameshift insertion	22.7
	*TP53*	c.799C>T	p.Arg267Trp	Nonsynonymous	21.1
	*JAK3*	c.2126G>T	p.Trp709Leu	Nonsynonymous	18.6
	*TLR4*	c.1325T>C	p.Val442Ala	Nonsynonymous	20.9
	*ATM*	c.8546G>T	p.Arg2849Leu	Nonsynonymous	20.9
	*ATM*	c.8549T>C	p.Leu2850Ser	Nonsynonymous	15.5
	*EPHA5*	c.2250G>A	p.Met750Ile	Nonsynonymous	19.2
	*TP63*	c.490G>T	p.Ala164Ser	Nonsynonymous	20.9
	*CRBN*	c.1020A>T	p.Leu340Phe	Nonsynonymous	23.2
	*TUBG1*	c.476A>C	p.Asp159Ala	Nonsynonymous	18.4
	*DAPK1*	c.754A>G	p.Ile252Val	Nonsynonymous	14
	*NOTCH4*	c.5186C>T	p.Ala1729Val	Nonsynonymous	19.2
	*TET2*	c.1842dup	p.Leu615AlafsTer23	Frameshift insertion	6.8
	*ANKRD11*	c.1041T>G	p.Tyr347Ter	Stopgain	5.7
MSI-H					
TMB high (13.62 mutations/MB)					

VAF: variant allele frequency; MSI: microsatellite instability; TMB: tumor mutational burden. Variants with VAF > 0.05 were shown.

Based on the dMMR/MSI-H/TMB-H phenotype of the patient, we suspected Lynch syndrome (LS) even though his personal and family history did not fulfill the revised Amsterdam criteria or Bethesda guideline (Cohen et al., 2019). Germline testing revealed a c.1144+1G>A mutation located at the splice donor site of intron 10 of the *PMS2* gene. This variant was not included in the InSiGHT database (http://www.insight-group.org/) (Thompson et al., 2014). In 2019, the two interpretations of this variant in the ClinVar database did not follow the 2015 ACMG–AMP guidelines (Richards et al., 2015) or the refined 2017 version (Sherloc) (Nykamp et al., 2017). To accurately determine its pathogenicity, we conducted a comprehensive genetic analysis. *In silico* analysis with three splice prediction programs including Alternative Splice Site Predictor (ASSP) (Wang and Marín, 2006), MaxEntScan (Yeo and Burge, 2004), and NetGene2 (Hebsgaard et al., 1996) suggested that the *PMS2* c.1144+1G>A variant will generate aberrant splicing transcripts (Table 2). Data in the gnomAD database revealed that its population allele frequency is 2/282496 without homozygotes, which falls into the pathogenic range defined by the Sherloc guideline (Nykamp et al., 2017). The proband had an oncogenic *KRAS* G13D somatic mutation and his 38-years-old daughter with the same *PMS2* variant had no cancer yet (Figure 1D). Moreover, Sanger sequencing results showed that this variant was segregated with his daughter but none of his five siblings (Figure 1E). We conducted an extensive literature review and found two probands harboring the same *PMS2* variant, a breast cancer patient from a cohort of 480 patients during a genetic screen of germline hereditary breast cancer susceptibility genes (Wang et al., 2018) and a colorectal cancer patient in a cohort of 6,503 patients from the NHLBI Exome Sequencing Project (ESP) (Amendola et al., 2015). According to the ACMG/Sherloc guidelines, we classified the *PMS2* c.1144+1G>A variant as class 4 (likely pathogenic). Interestingly, in a family with germline *PMS2* c.1144+2T>A variant, which disrupts the same splice donor site as c.1144+1G>A, the cosegregation between this variant and LS-related cancers was seen (Hendriks et al., 2006). These reports and our findings indicated that disruption of this specific *PMS2* splicing site can be pathogenic/likely pathogenic and result in LS.

**TABLE 2:** Prediction scores of reference sequence and c.1144+1G>A splicing variant in *PMS2* gene.

Tools	Exon intron junction sequence	
	Reference	c.1144+1G > A
	AAGgtaaga	AAGataaga
ASSP	10.959	-
MaxEntScan	10.57	2.39
NetGene2	0.99	-

Although the ESMO (2013), ASCO (2015), NCCN (2017) LS management guidelines gave the same recommendations for different MMR genes (Balmaña et al., 2013; Stoffel et al., 2015; Gupta et al., 2017), accumulating evidence demonstrated that the penetrance of *PMS2* was much lower than other MMR genes (Møller et al., 2017; Ten Broeke et al., 2018a; Ten Broeke et al., 2018b). Therefore, we provided *PMS2*-specific genetic counseling to the proband and the carrier. 5-yearly colonoscopic surveillance was recommended to the patient and the carrier. For the female carrier, endometrial/ovarian cancer screen was recommended after she reaches 50 years old. Furthermore, given the dMMR/MSI-H/TMB-H phenotype of the tumor and the recurrence risk of locally advanced NSCLC with concurrent *KRAS* and *TP53* mutations (Yagishita et al., 2015; Hames et al., 2016; Skoulidis and Heymach, 2019), the patient was then treated with cisplatin (75 mg/m^2^), pemetrexed (500 mg/m2), and nivolumab (200 mg) every 3 weeks for a total of 4 cycles after surgery for risk reduction. Sixteen months after the discontinuation of treatment, he developed progressive disease and was enrolled in a study with another investigational agent.

## Discussion

After the approval of pembrolizumab for patients with advanced dMMR/MSI-H solid tumor in 2017, the testing of dMMR/MSI-H has become a common diagnosis approach for cancer patients (Evans et al., 2021). If dMMR/MSI testing results are positive, germline testing will be recommended for the diagnosis of Lynch syndrome (LS), which is essential for the optimal care for cancer patients and their family members at risk (Yurgelun and Hampel, 2018). Currently, the standard of dMMR and MSI testing is the immunochemistry (IHC) assay of four MMR proteins and PCR-based assays of five microsatellite loci, respectively (André et al., 2020). One interesting observation in MMR IHC testing is that patients with pathogenic *MSH2* (*path*_*MSH2*) and *MLH1* (*path*_*MLH1*) mutations display simultaneous loss-of-expression for MSH2/MSH6 and MLH1/PMS2, respectively (Luchini et al., 2019). In contrast, patients with pathogenic *MSH6* (*path*_*MSH6*) and *PMS2* (*path*_*PMS2*) mutations retain expression of MSH2 and MLH1, respectively. This is because MSH6 forms a heterodimer complex with MSH2 and PMS2 forms a heterodimer complex with MLH1, which are required to maintain the protein stability of MSH6 and PMS2 but not MSH2 and MLH1, respectively. Therefore, the phenotypes of *path*_*MSH2* and *path*_*MLH1* single mutants mimic the phenotypes of *path*_*MSH2*/*path*_*MSH6* and *path*_*MLH1*/*path*_*PMS2* double mutants, respectively*.* According to these results, the cancer risk of *path*_*MSH2* and *path*_*MLH1* carriers should be significantly higher than *path*_*MSH6* and *path*_*PMS2* carriers*.*

This prediction was supported by multiple studies including the Prospective Lynch Syndrome Database (PLSD), an international, multicenter prospective observational study involving 6,350 *path_MMR* variants carriers and 1,808 observed cancers (Dominguez-Valentin et al., 2020). *Path*_*MSH2* and *path*_*MLH1* variants were associated with high penetrance dominant syndrome in colorectal, endometrial, and ovarian cancers while *path*_*MSH6* variants were associated with high risk in endometrial cancer but modestly increased risk for colorectal cancer. In contrast, the risk of *path*_*PMS2* variants for these three cancers was not increased before 50 years of age and only nonsignificantly increased after that.

The PLSD study series is a game changer for current cancer surveillance and risk-reduction practice for LS patients (Seppälä et al., 2021a; Dominguez-Valentin et al., 2021). According to the 2019 NCCN guideline for LS management, both the proband and carriers of *path*_*MMR* variants should take colonoscopy every 1–2 years for colorectal cancer surveillance and aspirin for risk reduction (Gupta et al., 2019). Additionally, the female carriers may consider an endometrial biopsy screen every 1–2 years for endometrial cancer surveillance and hysterectomy for risk-reduction. Given the incomplete penetrance of *PMS2* (Møller et al., 2017; Ten Broeke et al., 2018a; Ten Broeke et al., 2018b), we provided *PMS2*-specific genetic counseling to the proband and carrier, which was different from the general recommendation of the 2013 ESMO/2015 ASCO LS managenment guidelines (Balmaña et al., 2013; Stoffel et al., 2015). For instance, we did not recommend chemoprevention with aspirin or colonoscopy every one or two years for the proband and carrier. Our practice was largely consistent with the 2021 NCCN LS guideline which provided gene-specific cancer surveillance and risk reduction recommendations (NCCN Guideline, 2021). For instance, chemoprevention with 600 mg/daily aspirin for 2 years is recommended for all *path_MMR* carriers except for *path*_*PMS2* carriers. For *path*_*PMS2* and *path*_*MSH6* carriers, the colonoscopy screen age has been changed to 30–35 from 20–25 years old. Ovarian cancer screen or risk-reduction surgery are not recommended for *path*_*PMS2* carriers, as they do not have increased risk. In addition, the risk of endometrial cancer for *path*_*PMS2* carriers is only moderately increased compared to *path*_*MLH1, path*_*MSH2, and path*_*MSH6* carriers. Similarly, the 2021 LS guideline developed by the European Hereditary Tumour Group (EHTG) and the European Society of Coloproctology (ESCP) also revised the colorectal cancer surveillance and risk-reduction procedures for *path*_*MMR* carriers (Seppälä et al., 2021b). The colonoscopy screen interval time for *path*_*PMS2* and other *path*_*MMR* carriers are 5 years and 2–3 years, respectively. Moreover, the colonoscopy screen starting ages for *path*_*PMS2* and *path*_*MSH6* carriers are 35, but 25 for *path*_*MLH1* and *path*_*MSH2* carriers. Additionally, extended surgery is only recommended for *path*_*MLH1* and *path*_*MSH2* carriers but not for *path*_*PMS2* and *path*_*MSH6* carriers at the first diagnosis of colorectal cancer.

In addition to cancer surveillance and prevention, dMMR/MSI is also an important biomarker for cancer immunotherapy (Luchini et al., 2019). Due to the functional heterogeneity of MMR gene variants, sometimes we can see discordance between *path*_*MMR* variants and MSI status. This is well illustrated in the MSKCC pan-cancer MSI study of 15,045 patients including 103 LS cases (Latham et al., 2019). The microsatellite stable (MSS) cases in *path*_*PMS2*, *path*_*MSH6*, *path*_*MSH2*, and *path*_*MLH1* carriers were 68.2% (15/22), 53.8% (14/26), 13.9% (5/36), and 16.7% (3/18), respectively. This result indicates that the diagnosis of LS alone does not justify the treatment decision of immunotherapy, especially for *path*_*PMS2* and *path*_*MSH6* carriers.

Besides the discordance between *path*_*MMR* variants and MSI status, the discordance between dMMR and TMB is another issue oncologists should consider during the treatment decision-making process for LS patients. Recently, Bielska et al. reported that low TMB level in LS patients with dMMR tumors was a mechanism of immunotherapy resistance (Bielska et al., 2021). Three LS patients developed two primary dMMR tumors, one TMB-H and the other TMB-L. While the dMMR tumors with high TMB responded to immunotherapy, those with low TMB did not.

Next, we briefly discuss the limitations of TMB-H as the pan-cancer biomarkers for immunotherapy. In 2020, FDA approved PD-1 antibody pembrolizumab for the treatment of TMB-H (TMB >10 mutations/megabase) solid tumors. This approval was based on the results of the KEYNOTE-158 trial (Marcus et al., 2021). Pembrolizumab achieved an overall response rate (ORR) of 29% in TMB-H patients (13%, *n* = 102). However, this approval should not be applied to colorectal cancer (CRC) as subgroup analysis of 137 advanced CRC patients treated with immunotherapy showed that there is no survival benefit in TMB-H patients after the removal of patients with dMMR or *POLD*/*POLE1* mutations (Rousseau et al., 2021). Further pan-cancer analysis of 1,661 cancer patients treated with immunotherapy revealed that TMB-H was associated with improved overall survival in a limited subgroup of pMMR cancers including NSCLC (Rousseau et al., 2021). There are three important lessons that we can learn from these studies: first, TMB is not a pan-cancer immunotherapy biomarker; second, the combination of dMMR/MSI-H and TMB-H can provide better immunotherapy efficacy prediction than either alone; third, dMMR tumors with low TMB may not respond to immunotherapy.

LS is very rare in primary lung cancer. For instance, the MSKCC pan-cancer study did not find LS in 1,952 lung cancer patients including 94 MSI cases (Latham et al., 2019). We reviewed the literature and identified a few primary lung cancer cases associated with LS driven by *path*_*MSH2* (*n* = 4), *path*_*MLH1* (*n* = 1), *path*_*MSH6* (*n* = 1), and *path*_*PMS2* (*n* = 1) variants (Table 3). The *path*_*PMS2* lung cancer case was a 74-year old female non-smoker with MSS, pMMR, and TMB-L tumor (Sun et al., 2019). Without actionable mutations, she was treated with gefitinib for two months and then switched to platinum-based chemotherapy. The *path*_*MSH6* lung cancer case was a 76-year old male smoker with PD-L1-positive, pMMR, and TMB-L tumor (Long et al., 2021). Without actionable mutations, pembrolizumab was administered with SD at 4 cycles and PD at 8 cycles, likely due to an acquired *STK11* mutation. The *path*_*MLH1* lung cancer case was a 36-year old male non-smoker with dMMR/MSI-H tumor (Masuzawa et al., 2020). He received nivolumab as the fifth-line therapy for 15 cycles with a partial response lasting more than 20 months. Among the four *path*_*MSH2* lung cancer cases (Canney et al., 2009; Nolan et al., 2009; Kawashima et al., 2019; Sun et al., 2019), only one patient had dMMR/MSI tumor (Kawashima et al., 2019). He received nivolumab as the third-line therapy with a partial response lasting more than 10 months (Kawashima et al., 2019). Our patient was positive for three immune biomarkers (dMMR/MSI-H/TMB-H). These results suggested that he could benefit from immunotherapy.

**TABLE 3:** Clinical information and genetic testing results for seven lung cancer patients associated with LS.

Author, year	Histology	Age onset, y/Gender	Personal history (years)	Family history (years)	Germline mutation	TMB (muts/Mb)	MSI status	MMR expression	Genes with somatic mutations	Immuno-therapy
	Type											
S. Sun, et al., 2019	ADC	74/F	No	No	*PMS2* c.943C>T p.R315*	1	MSS	Intact	*PTCH1*	No
Y. Long, et al., 2021	SCC	76/M	No	Brother, gastric cancer; sister, colon cancer	*MSH6* c.2552_2553dupGC p.K852Afs*17	8	N/A	Intact	*ALK, LRP1B, TP53, DNMT3A, HRAS, DDR2, NTM, TCF7L2, POLE*	Yes
K.Masuzawa, et al., 2020	ADC	36/M	No	Colorectal cancer: sister (32 years), father (46 years, 54 years), paternal uncle (40 years), paternal grandmother (41 years)	*MLH1* c.2180_2181del p.H727Hfs*5	N/A	MSI-H	MLH1- MSH6-	N/A	Yes
A. Canney, et al., 2009	Two primary ADCs	59/M	Colorectal cancer (54 years, 58 years)	Father, leukemia died 70 years; brother, prostate cancer (62 years)	*MSH2* c.2109del p.I704Lfs*6	N/A	N/A	1st: MSH2- MSH6-; 2nd: intact	N/A	No
L. Nolan, et al., 2009	ADC	64/M	Colorectal cancer (33 years, 53 years); bladder cancer (51 years)	No	*MSH2* c.342+3C>T	N/A	MSI-H	MSH2-	N/A	No
Y. Kawashima, et al., 2019	ADC	68/M	Colorectal cancer (43 years, 63 years, 64 years); SCC (63 years); prostate cancer (67 years)	Mother, uterine cancer; brother, stomach cancer at a young age	*MSH2* rearrangement	N/A	MSI-H	MSH2- MSH6-	N/A	Yes
S. Sun, et al., 2019	ADC	62/F	No	Mother, colon cancer	*MSH2* c.340delG p.E114Rfs*60	1	N/A	Intact	*MAP2K2*, *GNAS*	No

M, male; F, female; ADC, adenocarcinoma; SCC, squamous-cell carcinoma; TMB, tumor mutational burden; MSI, microsatellite instability; MSS, microsatellite stable; N/A, not available.

Despite complete surgical resection, stage III NSCLC patients have high rates of relapse (Evison, 2020). Given the success of PD(L)-1 checkpoint inhibitors in metastatic NSCLC treatment, multiple trials are testing their efficacy in earlier stages of disease. Recently, results of the phase 3 IMpower010 trial showed a disease-free survival benefit with atezolizumab versus best supportive care after adjuvant chemotherapy in patients with PD-L1-positive resected stage II–IIIA NSCLC (Felip et al., 2021). This led to the FDA approval of atezolizumab as the adjuvant therapy for this patient population. Similarly, in the phase 2 KEYNOTE-799 trial, pembrolizumab plus concurrent chemoradiation therapy demonstrated objective response rates of 71% in locally advanced, stage III NSCLC (Jabbour et al., 2021). Because our case was a locally advanced dMMR/MSI-H tumor, we were also interested in the efficacy of adjuvant immunotherapy in this setting. For dMMR/MSI-H solid tumors, results of the phase 3 KEYNOTE-177 trial established that first-line pembrolizumab therapy resulted in significantly longer PFS than chemotherapy for dMMR/MSI-H metastatic CRC (André et al., 2020). Currently, two ongoing randomized phase 3 trials are testing the efficacy of adjuvant immunochemotherapy in patients with resected dMMR/MSI stage III CRC. The ATOMIC trial (NCT02912559) and the POLEM trial (NCT03827044) are evaluating the combination of chemotherapy with atezolizumab or avelumab, respectively (Sinicrope et al., 2019; Lau et al., 2020). Results of these two trials will provide proof-of-concept for the use of immune checkpoint inhibitors in the curative setting of dMMR/MSI-H stage III CRC which could be extended to other LS-associated solid tumors.

In summary, we encountered a locally advanced lung cancer patient with untargetable driver mutations, dMMR/MSI-H/TMB-H tumor, and a germline *PMS2* splicing variant which led to the diagnosis of LS. We provided *PMS2*-specific genetic counseling to the proband and the carrier in his family. The proband received 4 cycles of nivolumab plus chemotherapy for cancer-risk reduction, which led to a disease-free survival time of 16 months. Further efforts are required to investigate the efficacy of adjuvant immunotherapy in LS patients with locally advanced dMMR/MSI-H/TMB-H tumors.

## Data Availability

The original contributions presented in the study are included in the article/Supplementary Material, further inquiries can be directed to the corresponding authors.
