# Supplementary material for: Case Report and Literature Review: Diagnosis, Tailored Genetic Counseling and Cancer Prevention for a Locally Advanced dMMR/MSI-H/TMB-H Lung Cancer Patient With Concurrent Lynch Syndrome Mediated by a Rare PMS2 Splicing Variant (c.1144+1G>A)
Source: Front Genet. 2022 Jan 18;12:799807. doi: 10.3389/fgene.2021.799807 (PMC8804326; doi:10.3389/fgene.2021.799807)
Supplement: Supplementary file 1 [file Table1.pdf]

CARE case report guidelines

CARE Checklist of information to include when writing a case report

(cc) BY-NC-ND

Topic	Item No	Checklist item description	Reported on Page Number/Line Number	Reported on Section/Paragraph
Title	1	The diagnosis or intervention of primary focus followed by the words "case report"	Page 1 / Line 1-3	Title / Paragraph 1
Key Words	2	2 to 5 key words that identify diagnoses or interventions in this case report, including "case report"	Page 1-2 / Line 24-25	Key words / Paragraph 1
Abstract (no references)	3a	Introduction: What is unique about this case and what does it add to the scientific literature?	Page 3 / Line 29-42	Abstract / Paragraph 1
	3b	Main symptoms and/or important clinical findings	Page 3 / Line 38-41	Abstract / Paragraph 1
	3c	The main diagnoses, therapeutic interventions, and outcomes	Page 3 / Line 41-45	Abstract / Paragraph 1
	3d	Conclusion—What is the main "take-away" lesson(s) from this case?	Page 3 / Line 36-45	Abstract / Paragraph 1
Introduction	4	One or two paragraphs summarizing why this case is unique **(may include references)**	Page 3-5 / Line 48-78	Introduction / Paragraph
Patient Information	5a	De-identified patient specific information	Page 5 / Line 81	Presentation / Paragraph 1
	5b	Primary concerns and symptoms of the patient	Page 5 / Line 82-83	Presentation / Paragraph 1
	5c	Medical, family, and psycho-social history including relevant genetic information	Page 5-6 / Line 83-96	Presentation / Paragraph
	5d	Relevant past interventions with outcomes	Page 7 / Line 123-130	Presentation / Paragraph 3
Clinical Findings	6	Describe significant physical examination (PE) and important clinical findings	Page 5 / Line 83-86	Presentation / Paragraph 1
Timeline	7	Historical and current information from this episode of care organized as a timeline	Page 7 / Line 123-130	Presentation / Paragraph 3
Diagnostic Assessment	8a	Diagnostic testing (such as PE, laboratory testing, imaging, surveys).	Page 5 / Line 83-92	Presentation / Paragraph 1
	8b	Diagnostic challenges (such as access to testing, financial, or cultural)	NA	
	8c	Diagnosis (including other diagnoses considered)	Page 7 / Line 117-119	Presentation / Paragraph 2
	8d	Prognosis (such as staging in oncology) where applicable	Page 5 / Line 88	Presentation / Paragraph 1
Therapeutic Intervention	9a	Types of therapeutic intervention (such as pharmacologic, surgical, preventive, self-care)	Page 7 / Line 126-128	Presentation / Paragraph 3
	9b	Administration of therapeutic intervention (such as dosage, strength, duration)	Page 7 / Line 126-128	Presentation / Paragraph 3
	9c	Changes in therapeutic intervention (with rationale)	NA	

Follow-up and Outcomes	10a	Clinician and patient-assessed outcomes (if available)	NA	
	10b	Important follow-up diagnostic and other test results	Page 7 / Line 129-130	Presentation / Paragraph 3
	10c	Intervention adherence and tolerability (How was this assessed?)	NA	
	10d	Adverse and unanticipated events	NA	
Discussion	11a	A scientific discussion of the strengths AND limitations associated with this case report	Page 7-11 / Line 133-219	Discussion / Paragraph 1-6
	11b	Discussion of the relevant medical literature **with references**	Page 11-12 / Line 220-243	Discussion / Paragraph 7
	11c	The scientific rationale for any conclusions (including assessment of possible causes)	Page 12-13/ Line 244-264	Discussion / Paragraph 8
	11d	The primary "take-away" lessons of this case report (without references) in a one paragraph conclusion	Page 13-14 / Line 265-272	Discussion / Paragraph 9
Patient Perspective	12	The patient should share their perspective in one to two paragraphs on the treatment(s) they received	NA	
Informed Consent	13	Did the patient give informed consent? Please provide if requested	**Yes** ☑	**No** ☐

Please leave this space alone as it will be supplemented by the editorial office when needed.

Updated on April 13, 2020
